# Microwave-assisted extraction of pectin from grape pomace

**DOI:** 10.1038/s41598-022-16858-0

**Published:** 2022-07-26

**Authors:** Mariana Spinei, Mircea Oroian

**Affiliations:** Faculty of Food Engineering, Stefan Cel Mare University of Suceava, Suceava, Romania

**Keywords:** Polysaccharides, Polymer chemistry

## Abstract

The utilization of microwave technique for the pectin extraction from grape pomace (Fetească Neagră and Rară Neagră), its influence on yield, galacturonic acid content, degree of esterification and molecular weight of pectin were analyzed. The optimal conditions of the extraction process were microwave power of 560 W, pH of 1.8 for 120 s. The pectin samples extracted by MAE in optimal conditions were analyzed by comparing with commercial apple and citrus pectin based on FT-IR analysis, thermal behavior, rheological characteristics and microstructure. The FT-IR analysis established the presence of different functional groups which are attributed to the finger print region of extracted pectin, while the rheological behavior presented a good viscoelasticity of pectin solutions. The obtained data assumes that grape pomace has a great potential to be a valuable source of pectin which can be extracted by simple and quick techniques, while maintaining analogous quality to conventional sources of pectin.

## Introduction

Pectin is a family of natural polysaccharides of α-*D*-(1,4) galacturonic acid (GalA) present which are found in the plant cell walls (CWs) mostly in fruits, such as citrus fruits and apples, etc. and vegetables^1,2^. Besides, the main sources of pectin, other agricultural food by-products such as cocoa pod husks^3,4^, tomato waste^5^, potato pulp^6^, watermelon rind waste^7,8^, persimmon peel^9^, grape pomace^10,11^ were used for extraction, and the obtained pectin yield and its structural characteristics were investigated and stated. According to the regulation of Food and Agriculture Organization of the United Nations (FAO), pectin must contain more than 65% GalA, to be considered as being „desirable” in terms of quality^12^.

Usually, the production of pectin includes extraction, purification and drying. One of the most significant factors which influence the yield and quality of pectin is the use of a suitable method of extraction^13^. In the industry, the most commonly technique for the pectin extraction is conventional method which implicates the use of hot water (60–100 °C) acidified to a pH 1.5–3.0^14,15^, but different alternative methods have been implemented to maximize the pectin yield, such as enzyme-assisted^16,17^, microwave-assisted^18,19^, subcritical fluid^20,21^ and ultrasound pulses^17,19^.

The microwave-assisted extraction (MAE) is a technique which combines the use of microwave energy with that of a heat solvent to partition analytes from various sample matrices^22^, presenting a lot of advantages, such as high efficiency, low energy consumption, short processing time, low cost, cleanliness, easy controllability and low solvent requirements^19^. MAE enhances the water absorption capacity and capillary-porous components of the plant CW^23,24^; these modification contribute an opportunity to increasing the extraction yield of different analytes from plant CW, such as cellulose, hemicellulose and pectin^23^. Moreover, the energy causes the vibration of polar molecules with accelerated enhance of temperature which increases the extraction yield^25^. MAE has shown more efficient for the pectin extraction compared with conventional methods^14,25^.

In this study, pectin was extracted from grape pomace of two varieties, Fetească Neagră and Rară Neagră (*Vitis vinifera* L.), by applying microwave technique. This is the first research about utilization of a non-conventional extraction method, i.e. microwave-assisted extraction, to extract pectin from this by-product. The obtained pectin was described in terms of yield, galacturonic acid content, molecular weight and degree of esterification. Therefore, the aim of this study was to optimize the extraction process, to comprehend the effect of the working parameters on the yield and physicochemical characteristics of grape pomace pectin and correlated with those of commercial apple and citrus pectin.

## Materials and methods

### Materials

Grape pomace was collected by processing two different *Vitis vinifera* L. varieties (Fetească Neagră and Rară Neagră) from 2019 harvest, cultivated in the Bugeac area, Republic of Moldova. The grape pomace was dried in an oven at 50 °C until constant weight, then it was powdered and sieved in order to obtain 125–200 µm of particle size.

### Methods

All methods were performed in accordance with the relevant guidelines and regulations.

#### Extraction of pectin from dried grape pomace using MAE

MAE was performed in a household microwave oven Gorenje MO 17 DW (Gorenje, Velenje, Slovenia) at working frequency of 2450 MHz with regulable time and microwave power. 10 g of dried grape pomace was placed into bottle and ultrapure (Milli-Q) water containing different pH values (1, 2 and 3) at s solid–liquid ratio of 1:10 (w/v). The pH of the solvent was adjusted with citric acid. The bottle was fixed in the middle of the oven over a rotating dish and was subjected to microwave radiation at different powers (280, 420 and 560 W) for the selected exposure time (60, 90 and 120 s). After extraction procedure, the mixtures were allowed to cool down to room temperature (25 °C) and separated by centrifugation (35 min at 3500 rpm), followed by the precipitation procedure with an equal volume of 96% (v/v) ethanol. Finally, pectin was purified with 96% (v/v) ethanol for three times and dried to a constant weight at 50 °C in an oven.

#### Pectin yield

Pectin yield was determined using Eq. (1):

1 $$Yield \;(\% ) = \frac{{m_{0} }}{m} \times 100$$

where $m_{0}$, weight of dried pectin (g); $m$, weight of dried grape pomace powder (g)^18^.

#### Galacturonic acid content

The galacturonic acid (GalA) content of samples was measured using the sulfamate/*m*-hydroxydiphenyl method developed by Filisetti-Cozzi^26^ and Melton & Smith^27^. Sample preparation was made according to Miceli-Garcia^28^ and Dranca & Oroian^29^. The absorbance for each sample was read at 525 nm against the reageant control with a UV-3600 Plus UV–Vis–NIR spectrophotometer (Shimadzu Corporation, Kyoto, Japan).

#### Degree of esterification

The degree of esterification (DE) of samples was determined by the titrimetric method described by Franchi^30^ and Wai et. al.^31^. The DE was calculated with the Eq. (2):

2 $$DE \;(\% ) = \frac{{V_{2} }}{{V_{1} + V_{2} }} \times 100$$

where $V_{1}$, volume of sodium hydroxide used for the first titration (mL); $V_{2}$, volume of sodium hydroxide used for the second titration (mL).

The DE of pectin samples was measured in triplicate.

#### Molecular weight

Molecular weight (M_w_) of samples was carried out by high-performance size-exclusion chromatography using a HPLC system (Shimadzu Corporation, Kyoto, Japan) equipped with a LC-20 AD liquid chromatograph, SIL-20A auto sampler, a Yarra 3 µm SEC-2000 column (300 × 7.8 mm; Phenomenex, Torrance, CA, USA) and coupled with a RID-10A refractive index detector (Shimadzu Corporation, Kyoto, Japan). The samples were made according to Dranca et al.^18^.

#### Color

The color of the pectin samples was analyzed in triplicate at 25 °C with a CR-400 chromameter (Konica Minolta, Tokyo, Japan). CIE L*, hue (h*_ab_) and chroma (C*_ab_) were obtained from the reflection spectra of the pectin samples with illuminant D65 and 2° observer.

#### FT-IR analysis

The samples extracted by MAE in optimal conditions (FN and RN pectin) and the commercial samples (apple and citrus pectin) were conducted to to FT-IR analysis using a Spectrum Two infrared spectrophotometer (PerkinElmer, Waltham, MA, USA). The distinctive spectra were registered (three scans for each sample) in the frequency range of 4000–400 cm^−1^ at a resolution of 4 cm^−1^^32^.

#### Thermal analysis

Thermal analysis of pectin (samples extracted by MAE in optimal conditions, commercial apple and citrus pectin) was lead to differential scanning calorimetry technique (DSC); the measurement was repeated three times. DSC analysis of 1 mg dried pectin weighted in aluminum pan and placed in the apparatus (DSC 25, TA Instruments, New Castle, DE, USA) with an empty pan used as reference, were accomplished over a temperature range of 0–300 °C at a heating rate of 10 °C/min with a flow rate of 50 mL/min.

#### Rheological characterization of pectin solutions

In order to obtain 5% (w/w) solutions, pectin sample extracted by MAE in optimal conditions, commercial apple and citrus pectin were homogenized using Milli-Q water adjusted to pH 4 under constant stirring at 40 °C for 12 h. Then, the samples were cooled to room temperature (25 °C) and stocked under refrigeration at 4 °C for 16 h.

The dynamic viscosity of pectin solutions was determined with a Haake Mars 40 rheometer (Thermo Fisher Scientific, Waltham, MA, USA) using a cone (Ø 35 mm, 2°)—plate system at 20 °C. During measuring the dynamic viscosity (η, Pa·s) and shear stress (τ, Pa), the shear rate (${\dot{\gamma }}$, s^−1^) was ranged between 0 and 100 s^−1^. The stress sweeps of loss modulus (G′, Pa) and elastic modulus (G″, Pa) were determined at 1 Hz for measurement of the viscoelastic region. The frequency was presented a range from 0.1 to 100 Hz and the stress was selected within the linear viscoelastic region.

The ‘creep and recovery̕ analysis was measured at a constant stress of 1 Pa, which was implemented and maintained for 180 s; the stress was released to accept sample recovery for 180 s. The creep parameters were determined using Eq. (3):

3 $$J\left( t \right) = \frac{\gamma \left( t \right)}{\sigma }$$

where $J$, creep compliance; $\sigma$, constant stress over time ($t$); $\gamma$, shear deformation.

#### Microstructure

The structural morphology of the pectin samples was analyzed by scanning electron microscopy (SEM; SU-70, Hitachi, Tokyo, Japan). Powder of pectin was placed on the sample table with conductive double-sided adhesive carbon tape and analyzed using an accelerating voltage of 30 kV at different magnifications (150×, 400× and 700×).

#### Statistical analysis

In this sudy, a three-factor full factorial Box-Behnken design was adjusted in order to analyse and optimize the influence of the independent variables, power ($X_{1}$), pH ($X_{2}$) and time ($X_{3}$) on the extraction yield, DE, GalA content and M_w_ of pectin. The coded levels of the variables are presented in Table 1. All graphics and calculations were accomplished utilizing the statistical software Design Expert 13 (trial version, Minneapolis, MN, USA); the analysis was rehearsed in triplicate. The ANOVA test was used to evaluate the difference between means at the 95% confidence level (*p* < 0.05) with Fisher's least significant difference procedure.

**Table 1:** Variables and levels used for Box-Behnken design.

Variables	Levels		
	− 1	0	1
Microwave power (W)	280	420	560
Irradiation time (s)	60	90	120
pH	1	2	3

## Results and discussion

### Model fitting and statistical analysis

The microwave-assisted extraction (MAE) of pectin from Fetească Neagră (FN) and Rară Neagră (RN) grape pomace was modeled utilizing the Box-Behnken design with three parameters in accord with the data presented in Table 2. Each independent variable had three levels, as follows: microwave power (280, 420 and 560 W), irradiation time (60, 90 and 120 s) and pH (1, 2 and 3). The responses of the design were extraction yield (Y, %), galacturonic acid content (GalA, g/100 g), degree of esterification (DE, %) and molecular weight (M_w_, g/mol) of pectin.

**Table 2:** Box–Behnken experimental design matrix with measured and predicted values.

Run	Independent variables			Measured response				Predicted response			
	Microwave power (W)	Irradiation time (s)	pH	Y (%)	GalA (g/100 g)	DE (%)	M_w_ (g/mol)	Y (%)	GalA (g/100 g)	DE (%)	M_w_ (g/mol)
**FN**											
1	280	90	3	3.96	50.92	62.28	4.15 × 10^4^	3.66	48.04	61.89	4.11 × 10^4^
2	560	90	3	4.85	51.87	63.01	4.19 × 10^4^	4.77	52.12	63.82	4.17 × 10^4^
3	560	120	2	9.03	81.24	82.29	4.57 × 10^4^	8.61	77.93	80.74	4.53 × 10^4^
4	280	90	1	5.21	54.98	66.38	4.20 × 10^4^	5.29	54.73	65.57	4.21 × 10^4^
5	420	90	2	6.47	62.48	70.94	4.43 × 10^4^	6.79	66.30	72.90	4.46 × 10^4^
6	280	120	2	5.79	57.24	64.48	4.31 × 10^4^	5.58	57.07	68.13	4.29 × 10^4^
7	420	90	2	6.26	64.84	72.07	4.41 × 10^4^	6.79	66.30	72.90	4.46 × 10^4^
8	420	90	2	7.32	70.36	76.18	4.55 × 10^4^	6.79	66.30	72.90	4.46 × 10^4^
9	280	60	2	4.94	52.11	64.87	4.19 × 10^4^	5.36	55.42	66.42	4.23 × 10^4^
10	420	90	2	6.89	65.28	72.16	4.42 × 10^4^	6.79	66.30	72.90	4.46 × 10^4^
11	560	60	2	6.02	60.63	69.24	4.41 × 10^4^	6.23	60.80	69.59	4.43 × 10^4^
12	560	90	1	7.78	74.02	79.05	4.56 × 10^4^	8.08	76.91	79.44	4.59 × 10^4^
13	420	60	1	6.91	66.56	72.63	4.45 × 10^4^	6.40	63.50	71.89	4.40 × 10^4^
14	420	90	2	7.02	68.53	73.14	4.49 × 10^4^	6.79	66.30	72.90	4.46 × 10^4^
15	420	120	1	8.75	79.42	81.05	4.56 × 10^4^	8.88	79.84	82.21	4.56 × 10^4^
16	420	60	3	5.24	55.31	67.29	4.21 × 10^4^	5.11	54.71	66.13	4.21 × 10^4^
17	420	120	3	4.72	54.09	67.93	4.17 × 10^4^	5.23	57.15	68.67	4.22 × 10^4^
**RN**											
1	280	90	3	4.81	51.09	62.14	4.25 × 10^4^	4.47	49.04	60.99	4.21 × 10^4^
2	560	90	3	5.78	53.27	63.02	4.26 × 10^4^	6.16	53.09	63.21	4.24 × 10^4^
3	560	120	2	11.23	85.18	83.11	4.60 × 10^4^	10.26	81.71	81.45	4.56 × 10^4^
4	280	90	1	7.11	53.39	65.68	4.28 × 10^4^	6.72	53.57	65.48	4.31 × 10^4^
5	420	90	2	8.21	63.93	71.74	4.49 × 10^4^	8.42	67.85	72.94	4.51 × 10^4^
6	280	120	2	7.65	59.74	69.49	4.39 × 10^4^	7.41	58.13	69.18	4.37 × 10^4^
7	420	90	2	8.32	66.65	72.17	4.47 × 10^4^	8.42	67.85	72.94	4.51 × 10^4^
8	420	90	2	8.78	71.22	75.83	4.59 × 10^4^	8.42	67.85	72.94	4.51 × 10^4^
9	280	60	2	5.67	52.88	63.99	4.28 × 10^4^	6.64	56.35	65.64	4.31 × 10^4^
10	420	90	2	8.41	67.35	71.33	4.48 × 10^4^	8.42	67.85	72.94	4.51 × 10^4^
11	560	60	2	7.89	61.07	70.02	4.44 × 10^4^	8.13	62.67	70.32	4.46 × 10^4^
12	560	90	1	9.03	77.39	79.08	4.58 × 10^4^	9.37	79.44	80.22	4.62 × 10^4^
13	420	60	1	8.49	68.34	72.19	4.48 × 10^4^	7.90	64.69	70.73	4.42 × 10^4^
14	420	90	2	8.37	70.08	73.65	4.50 × 10^4^	8.42	67.85	72.94	4.51 × 10^4^
15	420	120	1	10.16	80.26	82.26	4.59 × 10^4^	10.79	81.68	82.76	4.59 × 10^4^
16	420	60	3	7.24	57.25	65.19	4.26 × 10^4^	6.61	55.83	64.68	4.27 × 10^4^
17	420	120	3	6.03	56.01	65.87	4.21 × 10^4^	6.62	59.66	67.32	4.27 × 10^4^

*FN* Fetească Neagră, *RN* Rară Neagră, *Y* yield, *GalA* galacturonic acid content, *DE* degree of esterification, *M*_*w*_ molecular weight.

The model applied to predict the evolution of the responses was a quadratic (second order) polynomial response surface model which was used to fit the results accomplished by design; the data of the analysis of variance (ANOVA) was presented in Table 3. The square polynomial equations that characterized the combined influence of microwave power ($X_{1}$), irradiation time ($X_{2}$) and pH ($X_{3}$) on extraction yield, GalA, DE and M_w_ are presented below.

4 $$\begin{aligned} Y_{FN} (\% ) & = 6.79 + 0.97 \cdot X_{1} + 0.64 \cdot X_{2} - 1.24 \cdot X_{3} + 0.54 \cdot X_{1} \cdot X_{2} - 0.42 \cdot X_{1} \cdot X_{3} \\ & \quad - 0.59 \cdot X_{2} \cdot X_{3} - 0.65 \cdot X_{1}^{2} + 0.30 \cdot X_{2}^{2} - 0.69 \cdot X_{3}^{2} \\ \end{aligned}$$

5 $$\begin{aligned} GalA_{FN} ({\text{g}}/100\;{\text{g}}) & = 66.3 + 6.56 \cdot X_{1} + 4.7 \cdot X_{2} - 7.87 \cdot X_{3} + 3.87 \cdot X_{1} \cdot X_{2} \\ & \quad - 4.52 \cdot X_{1} \cdot X_{3} - 3.47 \cdot X_{2} \cdot X_{3} - 4.67 \cdot X_{1}^{2} + 1.18 \cdot X_{2}^{2} - 3.68 \cdot X_{3}^{2} \\ \end{aligned}$$

6 $$\begin{aligned} DE_{FN} (\% ) & = 72.9 + 3.95 \cdot X_{1} + 3.22 \cdot X_{2} - 4.82 \cdot X_{3} + 2.36 \cdot X_{1} \cdot X_{2} \\ & \quad - 2.99 \cdot X_{1} \cdot X_{3} - 1.94 \cdot X_{2} \cdot X_{3} - 3.11 \cdot X_{1}^{2} + 1.43 \cdot X_{2}^{2} - 2.11 \cdot X_{3}^{2} \\ \end{aligned}$$

7 $$\begin{aligned} M_{w \;FN} \;({\text{g/mol}}) & = 44,635 + 1091.25 \cdot X_{1} + 419 \cdot X_{2} - 1302 \cdot X_{3} + 96.75 \cdot X_{1} \cdot X_{2} \\ & \quad - 803.25 \cdot X_{1} \cdot X_{3} - 369.75 \cdot X_{2} \cdot X_{3} - 827.88 \cdot X_{1}^{2} - 60.38 \cdot X_{2}^{2} - 1050.87 \cdot X_{3}^{2} \\ \end{aligned}$$

8 $$\begin{aligned} Y_{RN} (\% ) & = 8.42 + 1.09 \cdot X_{1} + 0.72 \cdot X_{2} - 1.37 \cdot X_{3} + 0.34 \cdot X_{1} \cdot X_{2} - 0.23 \cdot X_{1} \cdot X_{3} \\ & \quad - 0.72 \cdot X_{2} \cdot X_{3} - 0.8 \cdot X_{1}^{2} + 0.49 \cdot X_{2}^{2} - 0.93 \cdot X_{3}^{2} \\ \end{aligned}$$

9 $$\begin{aligned} GalA_{RN} ({\text{g}}/100\;{\text{g}}) & = 67.85 + 7.48 \cdot X_{1} + 5.21 \cdot X_{2} - 7.72 \cdot X_{3} + 4.31 \cdot X_{1} \cdot X_{2} \\ & \quad - 5.46 \cdot X_{1} \cdot X_{3} - 3.29 \cdot X_{2} \cdot X_{3} - 4.90 \cdot X_{1}^{2} + 1.78 \cdot X_{2}^{2} - 4.16 \cdot X_{3}^{2} \\ \end{aligned}$$

10 $$\begin{aligned} DE_{RN} (\% ) & = 72.94 + 4.24 \cdot X_{1} + 3.67 \cdot X_{2} - 5.37 \cdot X_{3} + 1.9 \cdot X_{1} \cdot X_{2 } \\ & \quad - 3.13 \cdot X_{1} \cdot X_{3} - 2.35 \cdot X_{2} \cdot X_{3} - 2.59 \cdot X_{1}^{2} + 1.3 \cdot X_{2}^{2} - 2.87 \cdot X_{3}^{2} \\ \end{aligned}$$

11 $$\begin{aligned} M_{w\; RN} ({\text{g/mol}}) & = 45,122.2 + 840.5 \cdot X_{1} + 407.88 \cdot X_{2} - 1193.38 \cdot X_{3} + 108.5 \cdot X_{1} \cdot X_{2} \\ & \quad - 716.5 \cdot X_{1} \cdot X_{3} - 415.25 \cdot X_{2} \cdot X_{3} - 622.73 \cdot X_{1}^{2} - 191.98 \cdot X_{2}^{2} - 1036.97 \cdot X_{3}^{2} \\ \end{aligned}$$

**Table 3:** Analysis of variance (ANOVA) of constructed quadratic model.

FN					RN				
Source	Sum of squares	Degree of freedom	Mean square	*F*-value	Source	Sum of squares	Degree of freedom	Mean square	*F*-value
**(A) Pectin yield (%)**									
Model	30.61	9	3.40	12.44**	Model	38.71	9	4.30	7.17*
MP	7.57	1	7.57	27.66**	MP	9.44	1	9.44	15.73**
IT	3.35	1	3.35	12.26*	IT	4.18	1	4.18	6.96*
pH	12.20	1	12.20	44.61**	pH	14.93	1	14.93	24.88**
MP × IT	1.17	1	1.17	4.26^ns^	MP × IT	0.46	1	0.46	0.77^ns^
MP × pH	0.70	1	0.70	2.58^ns^	MP × pH	0.22	1	0.22	0.37^ns^
IT × pH	1.39	1	1.39	5.09^ns^	IT × pH	2.07	1	2.07	3.46^ns^
R^2^ = 0.942					R^2^ = 0.902				
**(B) GalA (g/100 g)**									
Model	1365.79	9	151.75	10.95*	Model	1567.92	9	174.21	11.99*
MP	344.66	1	344.66	24.88**	MP	447.15	1	447.15	30.77**
IT	176.34	1	176.34	12.73*	IT	216.84	1	216.84	14.92*
pH	495.65	1	495.65	35.78**	pH	476.79	1	476.79	32.81**
MP × IT	59.91	1	59.91	4.32^ns^	MP × IT	74.39	1	74.39	5.12^ns^
MP × pH	81.81	1	81.81	5.91*	MP × pH	119.03	1	119.03	8.19*
IT × pH	48.30	1	48.30	3.49^ns^	IT × pH	43.30	1	43.30	2.98^ns^
R^2^ = 0.934					R^2^ = 0.939				
**(C) DE (%)**									
Model	534.71	9	59.41	15.80**	Model	628.89	9	69.88	18.41**
MP	124.66	1	124.66	33.15**	MP	143.91	1	143.91	37.92**
IT	82.69	1	82.69	21.99*	IT	107.60	1	107.60	28.35**
pH	186.24	1	186.24	49.53**	pH	231.02	1	231.02	60.88***
MP × IT	22.28	1	22.28	5.92*	MP × IT	14.40	1	14.40	3.80^ns^
MP × pH	35.64	1	35.64	9.48*	MP × pH	39.19	1	39.19	10.33*
IT × pH	15.13	1	15.13	4.02^ns^	IT × pH	22.04	1	22.04	5.81*
R^2^ = 0.953					R^2^ = 0.959				
**(D) M**_**w**_** (g/mol)**									
Model	3.57 × 10^7^	9	3.97 × 10^6^	11.18*	Model	2.79 × 10^7^	9	3.10 × 10^6^	9.08*
MP	9.53 × 10^6^	1	9.53 × 10^6^	26.85**	MP	5.65 × 10^6^	1	5.65 × 10^6^	16.51**
IT	1.40 × 10^6^	1	1.40 × 10^6^	3.96^ns^	IT	1.33 × 10^6^	1	1.33 × 10^6^	3.89^ns^
pH	1.35 × 10^7^	1	1.35 × 10^7^	38.22**	pH	1.14 × 10^7^	1	1.14 × 10^7^	33.28**
MP × IT	3.74 × 10^4^	1	3.74 × 10^4^	0.10^ns^	MP × IT	4.71 × 10^4^	1	4.71 × 10^4^	0.13^ns^
MP × pH	2.58 × 10^6^	1	2.58 × 10^6^	7.27*	MP × pH	2.05 × 10^6^	1	2.05 × 10^6^	6.00*
IT × pH	5.47 × 10^5^	1	5.47 × 10^5^	1.54^ns^	IT × pH	6.89 × 10^5^	1	6.89 × 10^5^	2.01^ns^
R^2^ = 0.935					R^2^ = 0.921				

ns, *p* > 0.05; *p < 0.01; **p < 0.001; ***p < 0.0001; FN, Fetească Neagră; RN, Rară Neagră; GalA, galacturonic acid content; DE, degree of esterification; M_w_, molecular weight; MP, microwave power; IT, irradiation time.

### Effect on process variables

#### Effect of extraction parameters on pectin yield

**Figure 1:**
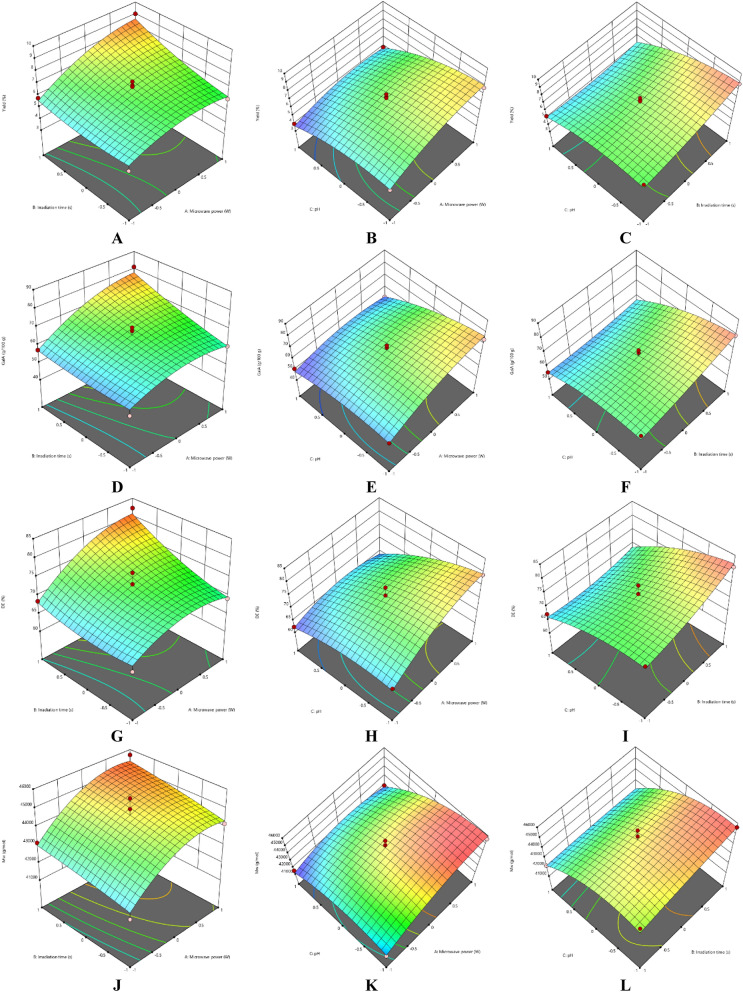
3D graphs showing the influence of extraction parameters on the yield (**A–C**), GalA content (**D–F**), DE (**G–I**) and M_w_ (**J–L**) for FN pectin.

**Figure 2:**
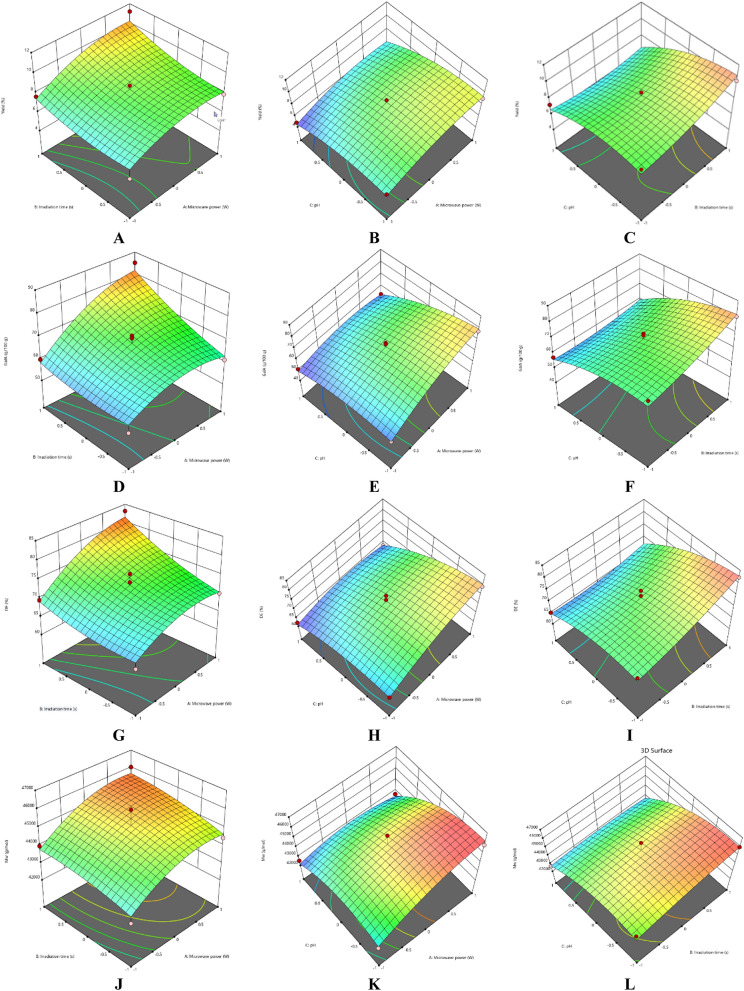
3D graphs showing the influence of extraction parameters on the yield (**A–C**), GalA content (**D–F**), DE (**G–I**) and M_w_ (**J–L**) for RN pectin.

The response surface methodology (RSM) plots (Figs. 1, 2) were used for the analysis of the influence of the independent variables on the pectin characteristics (extraction yield, GalA, DE and M_w_). For extraction yield of Fetească Neagră (FN) and Rară Neagră (RN) pectin, the three-dimensional graphics are shown in Figs. 1A–C, 2A–C, respectively. As the results of the ANOVA in Table 3 and 3D graphics present, that all the applied variables highly influenced the pectin yield. Extraction yield had a range between 3.96% (microwave power of 280 W, pH 3 for 90 s) and 9.03% (microwave power of 560 W, pH 2 for 120 s) for FN pectin, while for RN pectin, yield varied between 4.81% and 11.23% for similar extraction conditions.

The RSM plots showed the interaction effects of pH and microwave power, indicating that lower pH of solution led to higher yield of pectin when microwave power was 560 W. The similar phenomenon was identified when MAE was applied to extract pectin from apple pomace^22^, grapefruit^33^, navel orange peel^34^, sour orange peel^35^, papaya peel^36^ and banana peels^37^. This can be explained by the fact that during the propagation of the microwave power, the material absorbs the energy which result in enhanced heating of the solvent^13^. Moreover, the heat disrupts the cell walls (CWs) along with the diffusion of relevant compounds out of the material matrix^13^. Thus, the results proved that extraction yield of pectin increased as microwave power was enhanced; when the power level was enhanced (from 280 to 560 W), the solubility of pectin samples was increased with the effect of better extraction.

The second significant factor which influences the extraction yield of pectin during MAE was the treatment time. It was established that pectin yield was increased when the extraction time was enhanced up to 500 s^38,39^. On the other hand, Thirugnanasambandham et al.^40^ reported that the highest pectin yield extracted from dragon fruit peels was obtained at 400 W of microwave power, for 120 s at 45 °C and 24 g/mL of solid to liquid ratio. Also, for the extraction of pectin from sour orange peel, Hosseini et al.^41^ obtained the following optimal conditions in order to obtain the maximum pectin yield, 700 W of microwave power, for 180 s at pH 1.5. These results correlated well with the experimental data obtained in our study for the highest yield of pectin from grape pomace (FN and RN) extracted by MAE. Moreover, it could be concluded that MAE was a suitable treatment for extraction of pectin.

#### Effect of extraction parameters on galacturonic acid content

The influence of extraction characteristics on galacturonic acid (GalA) content of pectin extracted by MAE from FN and RN pomace are shown in Figs. 1D–F and 2D–F, respectively, while the results of the ANOVA are presented in Table 3. In accord with the values in Table 2, the highest GalA content was realized at the correlation of the following parameter values, microwave power of 560 W, pH 2 for 120 s (81.24 g/100 g and 84.18 g/100 g for FN and RN pectin, respectively), while the lowest GalA content (50.92 g/100 g and 51.09 g/100 g for FN and RN pectin, respectively) at the correlation among microwave power of 280 W, pH 3 and 90 s. Figures 1D and 2D indicate that GalA content enhanced with the microwave power, when irradiation time increased. The similar results were obtained when a high microwave power was applied to extract pectin from sour orange peel^35^, lime peel^25^ and lyophilized pumpkin flesh^42^. Therefore, it can be established that microwave heating may be an effective treatment in order to extract pectin from grape pomace without quality loss.

Figures 1E and 2E present that a higher GalA content were obtained when the pH value was low (less than 2). Thus, the interactions, microwave power-pH and extraction time–pH demonstrated a statistically significant effect on the GalA content of pectin samples extracted by MAE. The same data was achieved by Hosseini et al.^41^, they obtained the following optimal conditions: microwave power of 700 W, irradiation time of 180 s and pH 1.5 for the extraction of pectin from sour orange peel. On the other hand, Lefsih et al.^43^ reported that a range of pH from 1.5 to 3 didn’t affect the GalA content of pectin from *Opuntia ficus indica*. Moreover, the decrease of pH values, the GalA content and yield of pectin were found to decrease^43^. In addition, increasing the irradiation time (Figs. 1F, 2F) enables an enhancement of the GalA content from grape pomace pectin. The enhanced GalA content can be explained by the improved penetration in the plant matrix of microwaves during the extraction^44^. Similar results were reported for the sweet lemon peel pectin extracted by microwave under the optimal conditions (microwave power of 700 W, pH 1.5 and irradiation time of 180 s)^44^.

#### Effect of extraction parameters on degree of esterification

The degree of esterification (DE) is a significant characteristic for the determination of the pectin applications in the food industry, which is related to its texturizing, emulsifying and gelling properties^29,45^. The experimental and predicted values of DE are shown in Table 2, while the ANOVA results for DE are presented in Table 3. The correlations among microwave power, irradiation time and pH which establish the evolution of DE of the grape pomace pectin are illustrated in Figs. 1G–I, 2G–I. According to the results showed in Table 2, all pectin samples had a DE higher than 50%, ranging from 62.28% (microwave power of 280 W, pH 3 for 90 s) to 82.29% (microwave power of 560 W, pH 2 for 120 s) for FN pectin and a range of 62.14–83.11% for RN pectin under similar extraction conditions. The obtained data for DE of the samples presented same tendency in the evolution of the yield and GalA content of pectin.

As can be seen from Figs. 1G–I and 2G–I, the DE of pectin enhanced gradually with the increase of microwave power. At higher microwave power, temperature of the pectin solutions enhanced to improve the diffusion of different compounds. Therefore, the combined influence of irradiation time and microwave power (Figs. 1G, 2G) was more significant than other combined variables presented in Figs. 1I and 2I, as such a higher microwave power needs a shorter extraction duration in order to obtain a high value of DE and vice versa^46^. Some researchers suggest to use the approach of low power with longer irradiation time for extraction as high microwave power presented might reduce purity of pectin^37,46,47^. On the other hand, researchers prefer to use the treatment of high power with short irradiation time for pectin extraction^13,35,44,48^.

#### Effect of extraction parameters on molecular weight

The molecular weight (M_w_) of pectin is correlated with its gel-forming, thickening and stabilizing properties, which influence the utilization of pectin in the food industry^49^. Inadequate extraction conditions can affect the structure of pectin and thereby decrease its molecular weight^49,50^. For molecular weight of pectin from FN and RN pomaces, the 3D graphics are shown in Figs. 1J–L and 2J–L, respectively. The M_w_ of pectin samples ranged from 4.15 × 10^4^ g/mol (microwave power of 280 W, pH 3 for 90 s) to 4.57 × 10^4^ g/mol (microwave power of 560 W, pH 2 for 120 s) and 4.21 × 10^4^ g/mol (microwave power of 420 W, pH 3 for 120 s) to 4.60 × 10^4^ g/mol (microwave power of 560 W, pH 2 for 120 s) for FN and RN pectin, respectively (Table 2).

The microwave power and pH were significantly influenced the M_w_ values of pectin solutions (Figs. 1K, 2K); this can be explained by that pectin underwent more hydrolysis The same data was obtained by Li et al.^51^, they reported the following optimal conditions, 152.63 W of microwave power, pH 1.57 for 3.53 min and 18.92 solid to liquid ratio for sugar beet pulp pectin. Also, they stated that pH of solution had the highest influence on average M_w_ among the studied variables^51^. Moreover, Yoo et al.^42^ noted that pectin extracted by MAE at pH 2 had more than 5 times higher M_w_ than pectin extracted at pH 1. Microwave power implicates the conversion of high-frequency energy into heat energy which ensues in a more efficient pectin extraction^52^. On the other hand, Bagherian et al.^33^ reported that M_w_ decreased with the increasing of microwave power and heating time; they explained that continued heating may lead to pectin degradation (disaggregation of pectin matrix).

### Optimization and validation of extraction conditions

The desirability function approach was utilized to optimize pectin characteristics (extraction yield, GalA content, DE and M_w_) concurrently. The first characteristic ($y_{i}$) was converted into desirability function ($d_{i}$), which varied over the range presented in Eq. (12):

12 $$0 \le d_{i} \le 1$$

In order to achieve the highest extraction yield, GalA content, DE and M_w_ of pectin from grape pomace (FN and RN), microwave power, irradiation time and pH were optimized. The optimal conditions were the following, 560 W, pH 1.8 and 120 s which presented a desirability function of d = 0.852 and d = 0.861 for RSM plots of FN pectin (9.03% pectin yield, 81.24 g/100 g of GalA content, 82.29% DE and 4.57 × 10^4^ g/mol of M_w_) and RN pectin (11.23% pectin yield, 85.18 g/100 g of GalA content, 83.11% DE and 4.60 × 10^4^ g/mol of M_w_), respectively. The data was well correlated with the predicted values of responses, so the optimal conditions for MAE of pectin samples were valid.

### Color

The pectin color is a significant factor affecting the appearance of gel produced and then the characteristic of the food product in which was added^53^. As can be seen in Table 4, the commercial apple pectin had the highest lightness value (63.65), while the lowest lightness value (48.70) for RN pectin. The samples extracted by MAE, commercial apple and citrus pectin were characterized by more redness. In terms of color, commercial apple and citrus pectin (CAP and CCP) were similar in comparison with FN and RN pectin. Among them, CAP had the highest chroma (20.68) and hue (81.18). Also, the MAE treatment influenced significantly the chroma (C*_ab_) and hue (h*_ab_) of pectin samples. This can be explained by the fact that pectin extracted under higher power and temperature for prolonged time has a lower value of lightness (L*). Moreover, the color values of grape pomace pectin were predominantly due to tannins and anthocyanins which are the main polyphenolic compounds responsible for color in red grapes^54^. The similar tendency was reported for pectin extracted from lime peel^25^ and apple pomace^55^.

**Table 4:** Color characteristics, thermal properties, creep and recovery parameters of pectin samples. Mean values and standard deviation, in brackets.

Sample	Color characteristics			Peak integration		Creep and recovery parameters				
	L*	C*_ab_	h*_ab_	ΔH_d_ (J/g)	T_d_ (°C)	J_e_ (1/Pa)	J_r_ (1/Pa)	$\dot{\upgamma }$(1/s)	η (Pa·s)	d(log($\dot{\upgamma }$))/d(log(t)) (1/s)
FNP	62.17 (0.05)^a^	12.24 (0.10)^c^	39.10 (0.18)^c^	306.24 (0.12)^a^	276.73 (0.16)^a^	4.547 (0.23)^d^	0.682 (0.21)^c^	0.1252 (0.15)^a^	8.034 (0.27)^d^	0.907 (0.12)^a^
RNP	48.70 (0.02)^b^	17.97 (0.12)^b^	31.66 (0.23)^d^	212.94 (0.09)^b^	267.97 (0.22)^b^	0.993 (0.18)^b^	0.219 (0.16)^b^	0.0564 (0.24)^c^	17.810 (0.32)^b^	0.825 (0.04)^b^
CAP	63.65 (0.06)^a^	20.68 (0.08)^a^	81.18 (0.27)^a^	30.08 (0.17)^d^	217.88 (0.18)^d^	4.334 (0.16)^c^	0.773 (0.08)^d^	0.0902 (0.18)^b^	11.130 (0.24)^c^	0.782 (0.22)^c^
CCP	53.74 (0.11)^b^	12.49 (0.16)^c^	77.14 (0.21)^b^	45.80 (0.24)^c^	238.64 (0.21)^c^	0.037 (0.12)^a^	0.062 (0.07)^a^	0.0004 (0.12)^d^	2380 (0.29)^a^	0.662 (0.08)^d^
*F*-value	8.81*	47.26***	4692.87***	3.06 × 10^8^***	2.19 × 10^6^***	5.27 × 10^6^***	1.79 × 10^5^***	2.09 × 10^5^***	4.20 × 10^6^***	7.84 × 10^5^***

ns, *p* > 0.05, *p < 0.01, **p < 0.001, ***p < 0.0001, ^a–d^different letters in the same column indicate significant differences among samples (*p* < 0.0001) according to the LSD test with α = 0.05. FNP, Fetească Neagră pectin; RNP, Rară Neagră pectin; CAP, commercial apple pectin; CCP, commercial citrus pectin; L*, lightness of the color; C*_ab_, chroma; h*_ab_, hue angle; ΔH_d_, degradation enthalpy; T_d_, degradation temperature; J_e_, equilibrium compliance; J_r_, recoverable compliance; $\dot{\upgamma }$, shear rate; η, viscosity.

### FT-IR analysis

**Figure 3:**
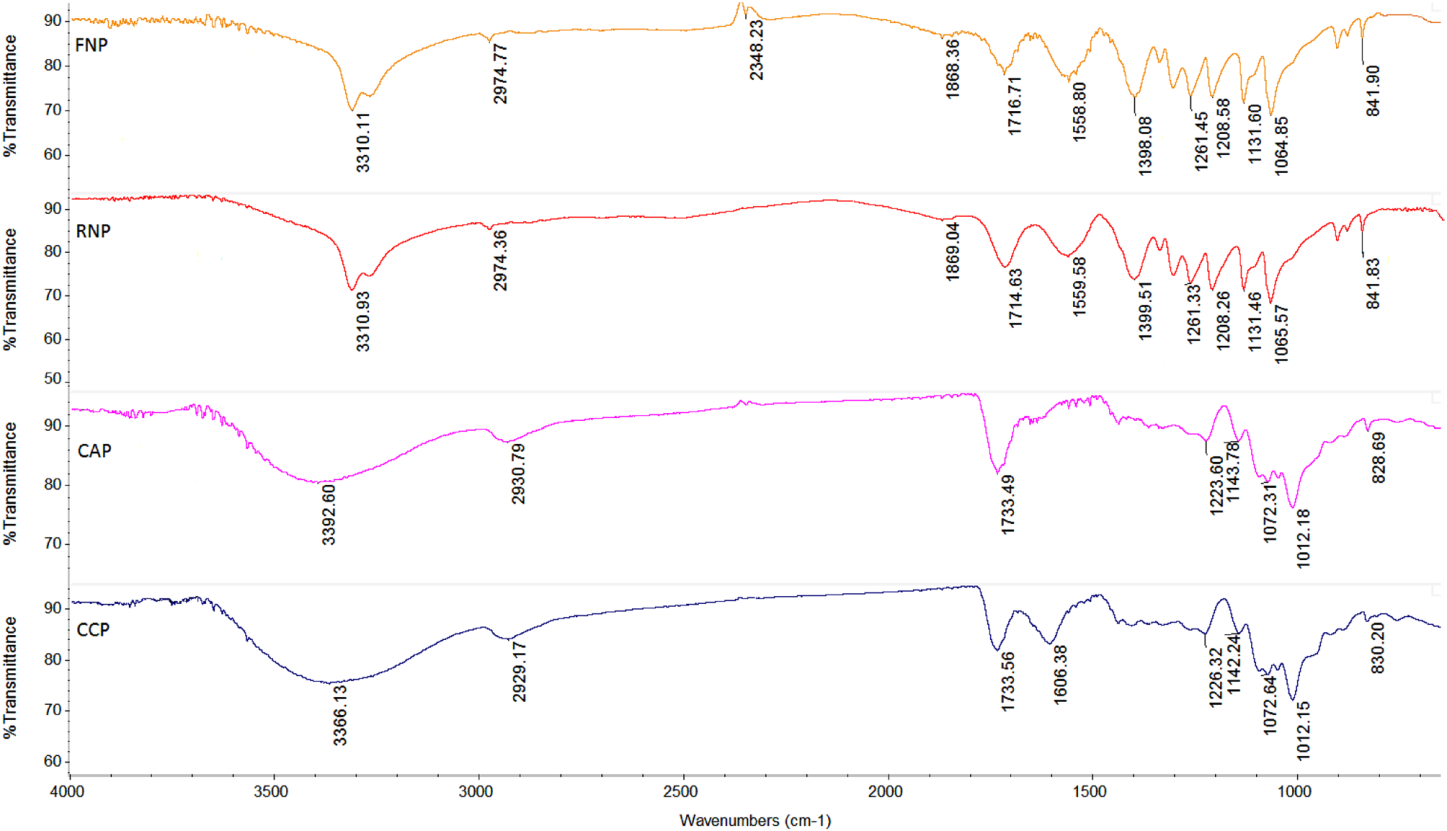
FT-IR spectra of pectin extracted from grape pomace (FN and RN) by MAE under the optimal conditions, commercial apple and citrus pectin (CAP and CCP).

The different structural particularities of pectin extracted from grape pomace pectin (FN and RN) by applying microwave technique and correlate them to two commercial pectin samples (apple and citrus), FT-IR analysis was utilized. The FT-IR spectra of commercial samples (apple and citrus pectin) and pectin obtained by MAE in the optimal conditions are presented in Fig. 3. By comparing the spectra, FN and RN pectin had a peak around 3310 cm^−1^ which was ascribed to intermolecular bonding of O(6)H···O(3)^56^, while citrus and apple pectin had a shift at 3392–3366 cm^−1^ which was attributed to –OH and carbonyl C=O stretching vibrations^57^. The absorption peaks at 2929–2974 cm^−1^ which were found in pectin samples (commercial and extracted by MAE), was related to –CH (CH, CH_2_ and CH_3_) vibrations^58,59^. The C–H stretching detected at the peak of 2348 cm^−1^ is characteristic for polysaccharides chains^60^, while the peak of 1868 cm^−1^ was corresponded to the symmetric and asymmetric C–O stretching^61^. The band positions at 1714 cm^−1^ (FN and RN pectin) and 1733 cm^−1^ (commercial apple and citrus pectin) were assigned to undissociated carboxylic acid (COOH) and –CO from the group methyl ester (COOCH_3_)^62^. The band identified at 1606 cm^−1^ was due to the asymmetric stretching vibration of the carboxylate ion (COO–) and C = C ring stretching of phenolic compounds^63^, while the peak at 1559 cm^−1^ was ascribed to the valence vibration of C = O bond^64^.

The absorption bands at 1399 cm^−1^ and 1261 cm^−1^ indicated enhancement quantity of carboxylates^65^ and –CH bending^66^, respectively. The FT-IR spectra in the wavenumber between 1300 and 800 cm^−1^ are referred to as the ‘fingerprint region’ of carbohydrates, which enables the identification of major chemical groups in different polysaccharides^29,67^. Therefore, the peaks at 1226 cm^−1^, 1223 cm^−1^ and 1208 cm^−1^ were assigned to the stretching vibration of C single bond, C = O stretching and C–O–H bending, respectively^25^. The peaks at around 1143 cm^−1^ and 1131 cm^−1^ were attributed to the C–O, C–O–C and C–C rings, which are characteristic to the structure of commercial (apple and citrus pectin) samples and pectin (FN and RN) extracted by MAE, respectively^68^. The bands identified at 1072 cm^−1^ (commercial apple and citrus pectin) and 1065 cm^−1^ (FN and RN pectin) were ascribed to C–O and C–C stretching of xyloglucan^69^ and galactoglucomannan^68^, respectively. Some peaks are more intense in isolated pectin, such as at 1012 cm^−1^ (C–O, C–C and C–O–H stretching)^69^, 842 cm^−1^ (CH_2_ bending linked to α-arabinose pyranoid ring)^68^, 828 cm^−1^ and 830 cm^−1^ (α-*D*-mannopyranose)^70^.

### Thermal analysis

**Figure 4:**
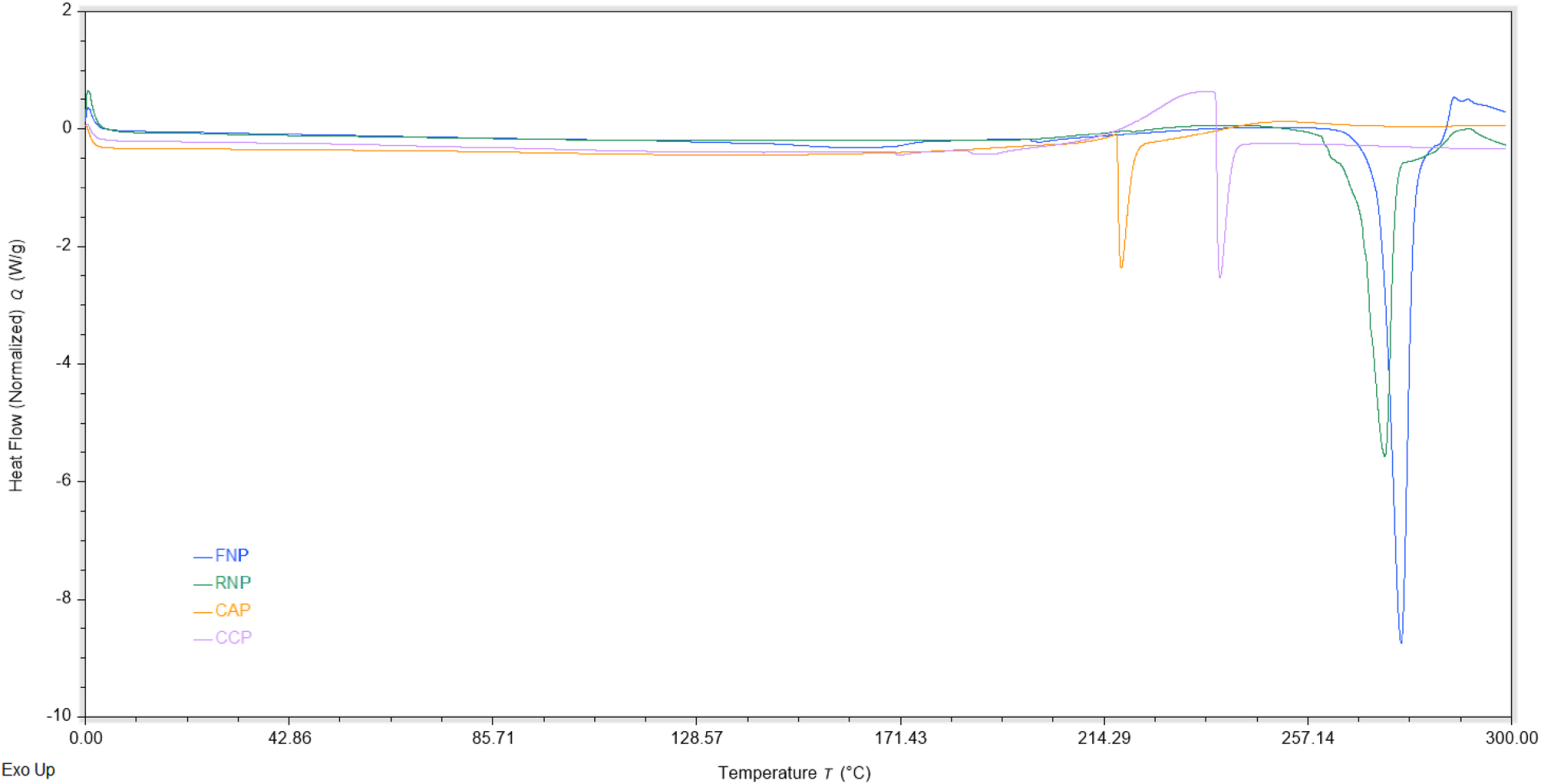
DSC thermograms of pectin extracted from grape pomace (FN and RN) by MAE under the optimal conditions, commercial apple and citrus pectin (CAP and CCP).

The DSC was employed to examine the thermal characteristics and describe changes during thermal denaturation of pectin samples (FN and RN pectin extracted by MAE, commercial apple and citrus pectin) as illustrated in Fig. 4 and Table 4. Generally, thermal properties of pectin depend on the interdependence of three factors, its state transition occurred during processing, chemical composition and stability properties^71,72^. In the thermogram of pectin samples, exothermic peaks (degradation temperature) were registered at temperatures between 217.88 and 276.73 °C, while endothermic peaks (melting temperature) were not noticed. The endothermic peaks appear from water evaporation, melting-recrystallization of the crystallites, hydrogen bonding of GalA units, and also a conformational change of the pectin pyranose rings^72–76^. In this study, the absence of endothermic peaks indicates that no bound water was removed from the pectin samples^18^.

Commercial apple and citrus pectin samples had peak temperatures ($T_{d}$) at 217.88 and 238.64 °C, respectively, and these results were identical to others established in the scientific studies for the same pectin sources^18,74,75^.The exothermic peaks for pectin samples extracted by MAE had following values: FN—276.73 °C and RN—267.97 °C with a degradation enthalpy ($\Delta H_{d} )$ of 306.24 J/g and 212.94 J/g, respectively. As it can be deduced, pectin obtained by MAE presented higher thermal stability than the commercial sources of pectin, which denotes that FN and RN samples might be preferred during thermal procedure. Therefore, $T_{d}$ of pectin samples was influenced by their composition, while $\Delta H_{d}$ was affected by GalA content^75^.

### Rheological properties

#### Flow behavior of pectin solutions

**Figure 5:**
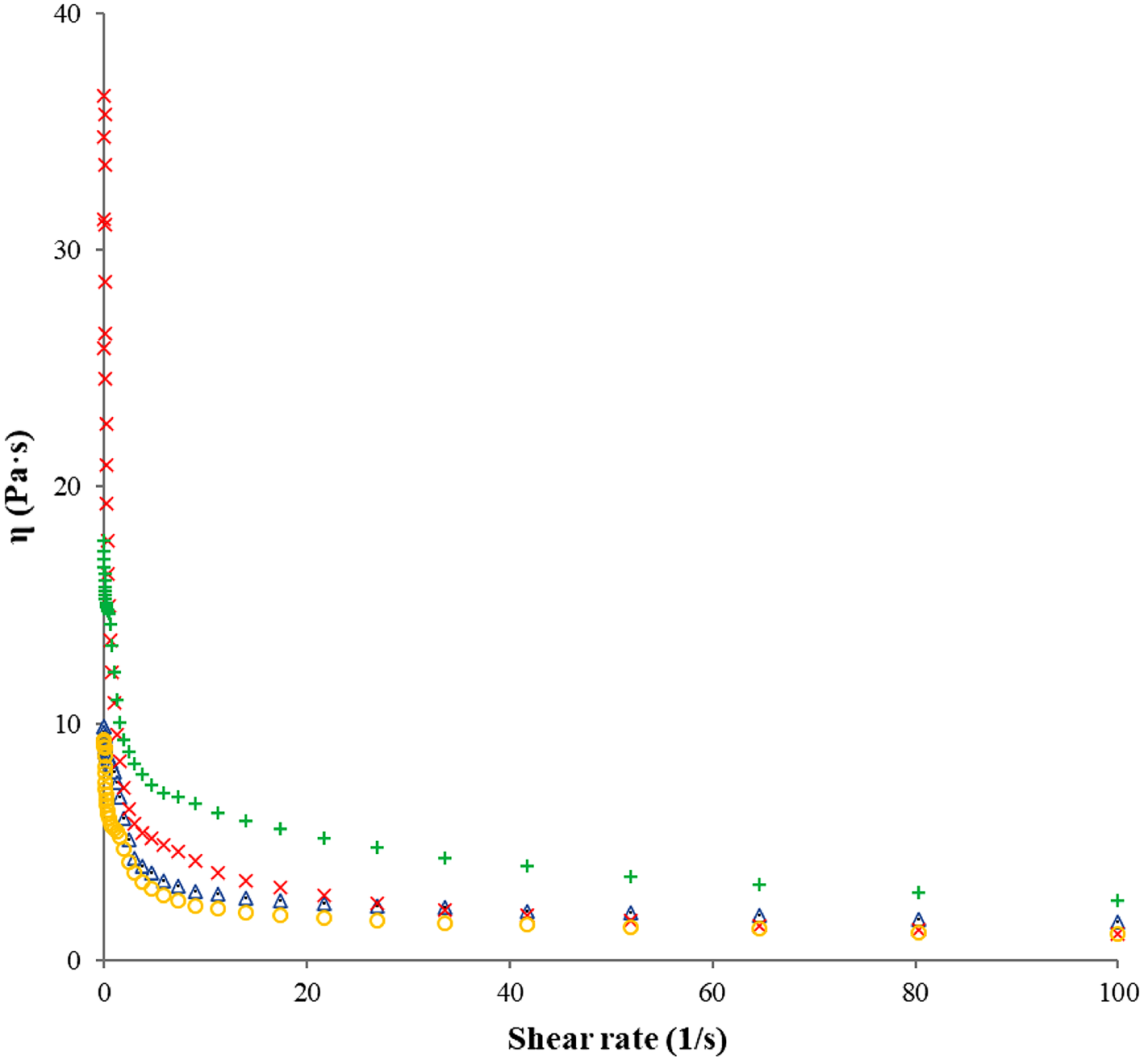
Flow curves of pectin solutions: Fetească Neagră pectin (**○**), Rară Neagră pectin (**+**), commercial apple pectin (Δ) and commercial citrus pectin (**×**); $\eta$—dynamic viscosity, $\dot{\gamma }$—shear rate.

The flow curves of the commercial pectin samples (apple and citrus) and pectin extracted by MAE (FN and RN) are presented in Fig. 5; all curves show a non-Newtonian fluid behavior with an enhancement in the shear stress and a decrease of the dynamic viscosity. This behavior was assigned to the decrease of the pectin intermolecular forces during the stress application^25,77^. It was noticed that RN pectin extracted by MAE had a higher dynamic viscosity than other samples, which means that source of pectin and different extraction parameters affect the pectin flow behavior^25,29^. The viscosity of RN pectin extracted by MAE at a shear rate of 100 s^−1^ was 2.53 Pa·s which was higher than viscosity of other samples, commercial citrus pectin (1.61 Pa·s), commercial apple pectin (1.11 Pa·s) and FN pectin extracted by MAE (1.01 Pa·s). Moreover, this value was also higher than the dynamic viscosity obtained at the same shear rate (100 s^−1^) for different concentration (0.5%, 1%, 2% and 3%) of lime peel pectin solution (less than 1 Pa·s)^25^, 1.5% and 2% pectin solutions of sour orange peel (less than 0.01 Pa·s)^44^ and 30 g/L pectin solution of finger citron pomace (0.5 Pa·s)^70^.

The viscosity of pectin solutions enhances with the increasing pectin concentration, while the intermolecular space between the pectin molecules reduce^78^; this behavior has been noticed for pectin from apple pomace^29,79^, citrus peel^80^, pomelo peels^46^, cacao pod husks^81^ and sour orange peel^41^.

#### Viscoelastic properties of pectin solutions

**Figure 6:**
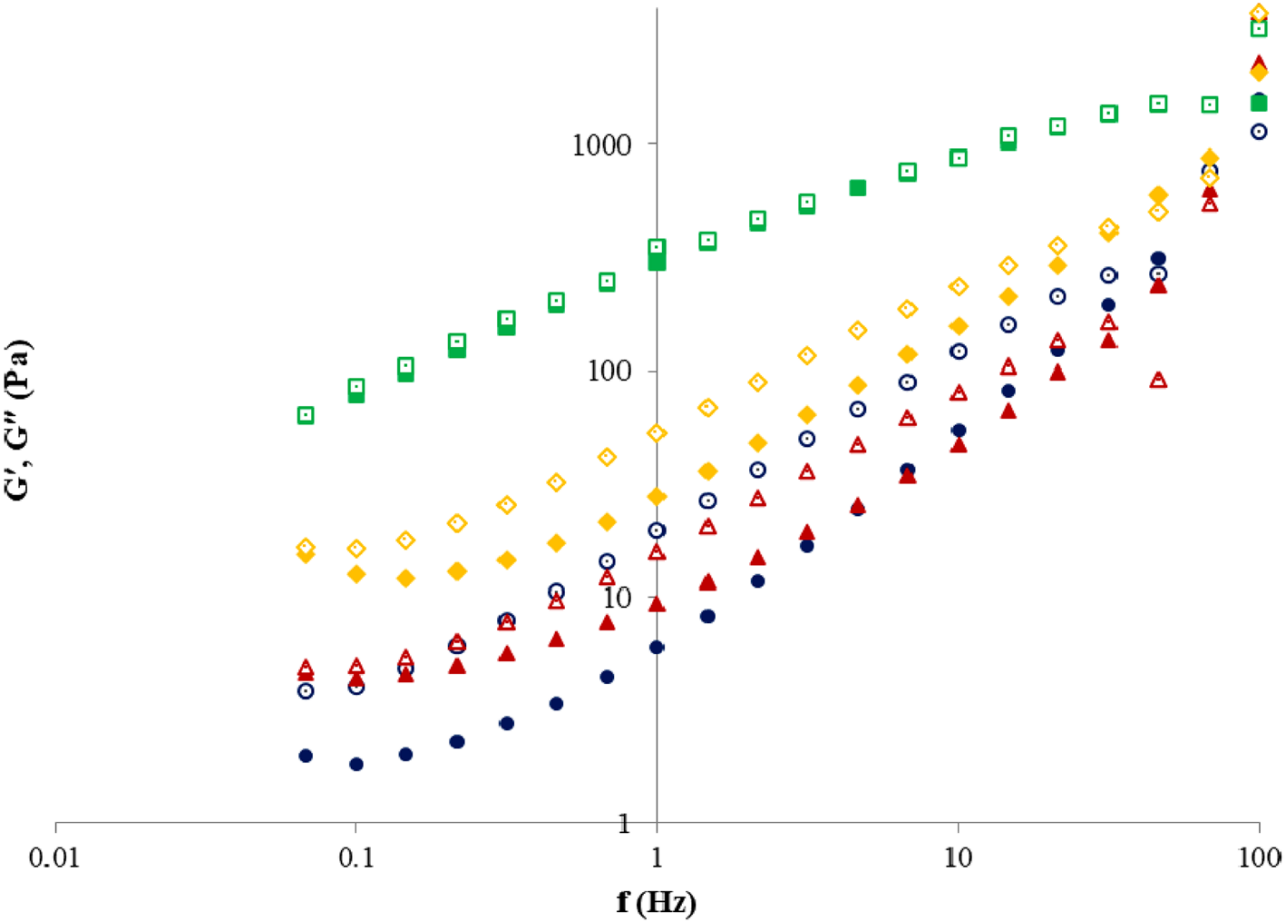
Elastic modulus (fill symbol) and loss modulus (no fill symbol) for different pectin samples: Fetească Neagră pectin (▲, ∆), Rară Neagră pectin (♦, **◊**), commercial apple pectin (●, **○**) and commercial citrus pectin (■, **□**); ${G}^{^{\prime}}$—elastic modulus, ${G}^{^{\prime\prime} }$—loss modulus, $f$—frequency.

Figure 6 shows the viscoelastic characteristics of the 5% pectin solutions; the elastic modulus (G′) and loss modulus (G″) were determined in the linear viscoelastic region. The elastic modulus (G′) serves as the elastic constituent of the stress, while the loss modulus (G″) determines the energy lost via viscous flow^82^. All pectin samples had a higher G″ (liquid behavior) than G′ (solid behavior) in the 0.1–100 Hz frequency domain applied (Fig. 6). The values of both moduli enhanced proportionally with the frequency. Therefore, the G′ enhances more sharply with frequency correlated to the behavior of G″, until the two curves intersect and elastic constituents override the viscous. For that reason, the ability of pectin network to keep the temporarily enforced energy enhances, and it involves more like an elastic solid^81^. Furthermore, the intersection of moduli (G′ and G″) shows the good viscoelasticity of pectin solutions^83^; the lower the value of intersection moment, the major the role of elasticity^84^. The similar behavior was also noticed for the 5% pectin solutions from cacao pod husks^81^, lime peel waste^25^, pulp of gabiroba^85^ and apple pomace^79^. In addition, the extraction methods had a considerable impact on the rheological parameters of pectin and samples extracted by microwave treatment could be considered adequate for using in diverse food products.

#### ‘Creep and recovery̕ analysis

**Figure 7:**
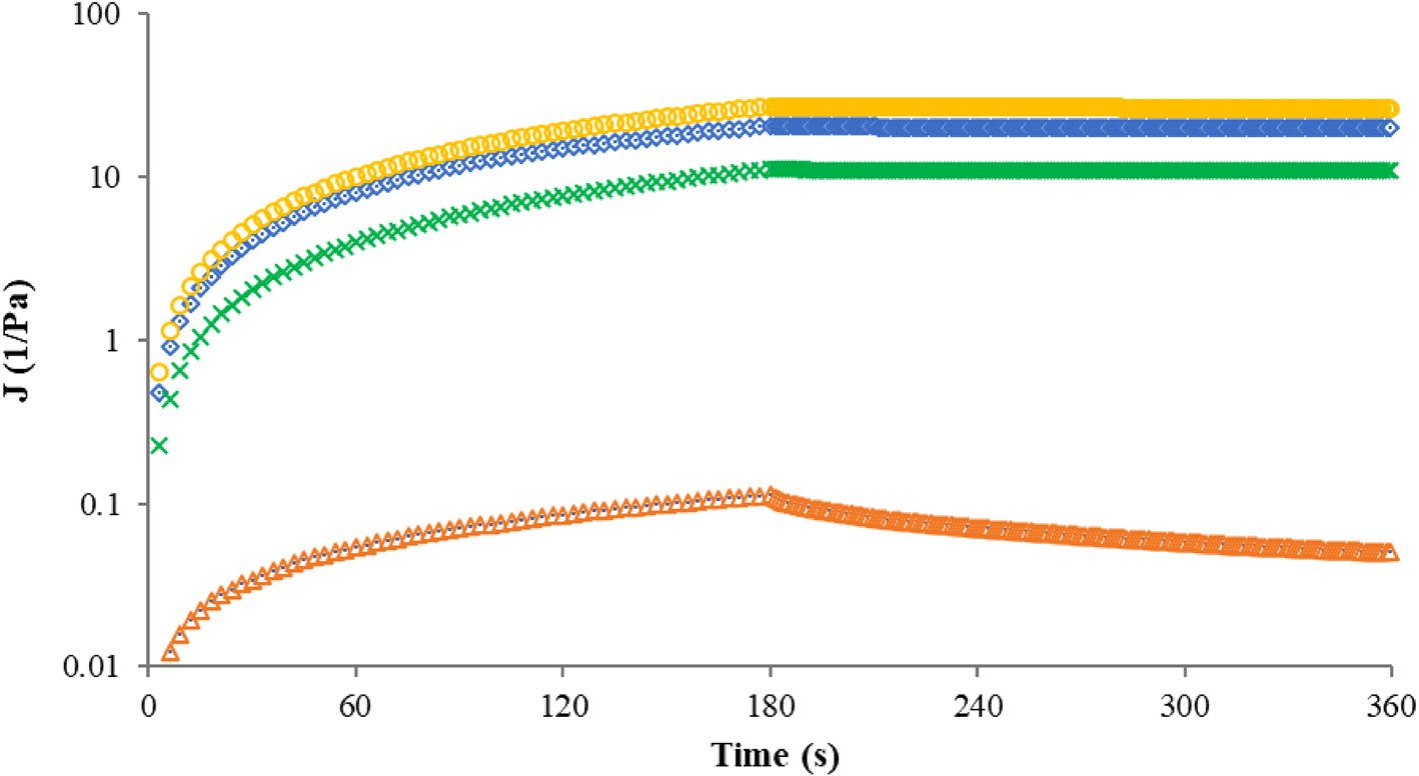
Creep and recovery test of pectin solutions: Fetească Neagră pectin (**○**), Rară Neagră pectin (**×**), commercial apple pectin (**◊**) and commercial citrus pectin (**∆**); $J$—compliance.

In the ‘creep and recovery̕ analysis (Fig. 7), the pectin samples were subjected to a constant stress during 360 s in order to assess the material deformation; the ‘creep̕ test is from 0 to 180 s, while the ‘recovery̕ test is from 180 to 360 s. The ‘recovery̕ analysis allows access to the rheological behavior of material and lower shear rates for various systems^86^. The ‘creep and recovery̕ analysis, through the creep compliance ($J$) as time function, distinguishes between the viscous and elastic phases. In viscoelastic materials, ‘recovery̕ phase of the applied stress is partial, controlled by viscous or elastic characteristic of the samples, established at a transitional point between liquid and solid^86^. All samples (FN and RN pectin extracted by MAE; commercial apple and citrus pectin) manifested a non-Newtonian behavior, with a decrease of strain response during the applied stress, evidencing their viscoelastic characters. Commercial citrus pectin solution had a better recovery than other pectin samples. The creep and recovery parameters are presented in Table 4; the equilibrium and recoverable compliance ($J_{e}$ and $J_{r}$), respectively) values for commercial citrus pectin and RN pectin were lower than commercial apple pectin and FN pectin. The highest shear rate ($\dot{\gamma }$) was observed for FN pectin (0.1252 1/s), while the lowest was obtained for CCP (0.0004 1/s); the similar tendency was noticed for d(log($\dot{\gamma }$)/d(log(t)).

### Microstructure

**Figure 8:**
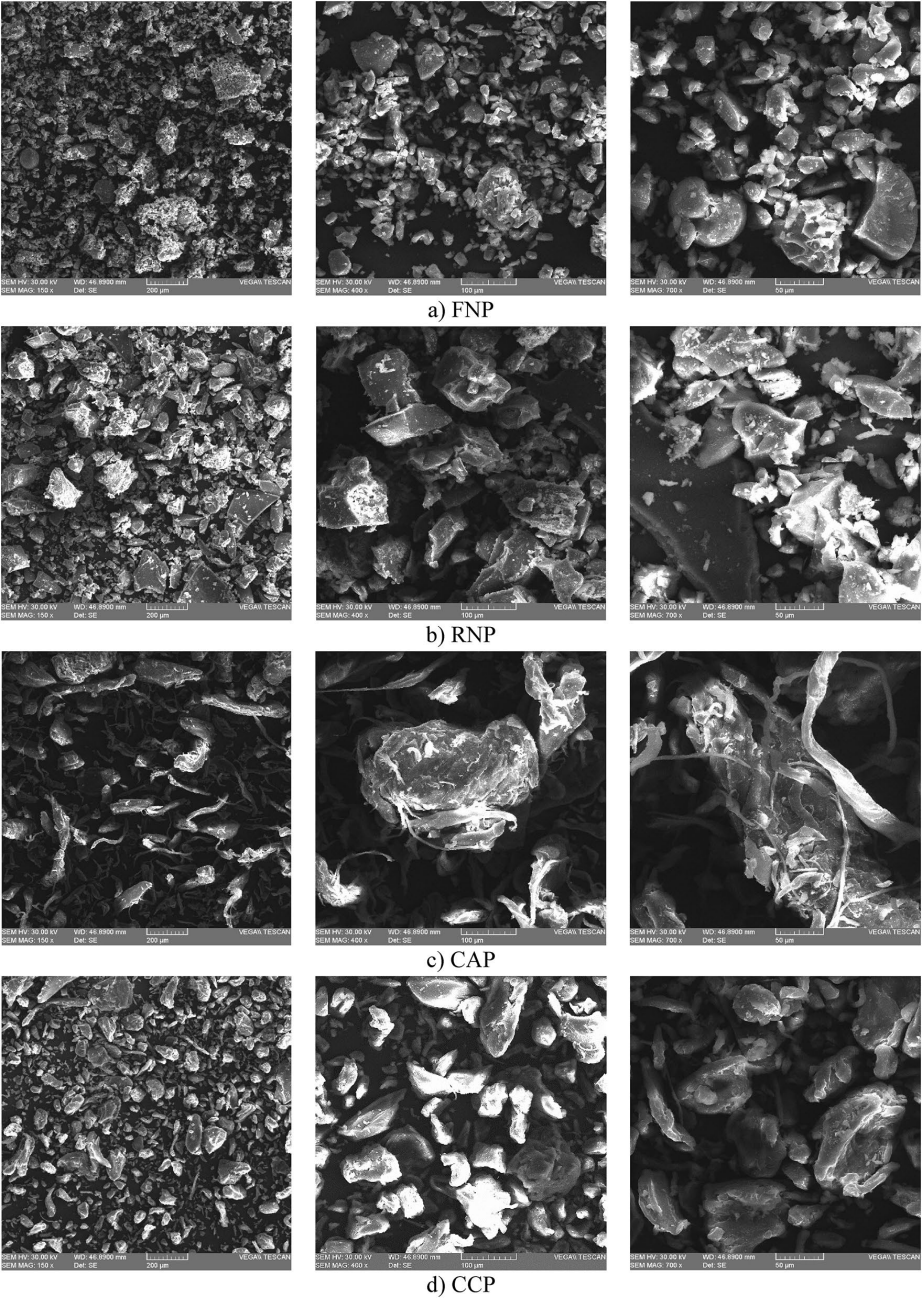
SEM images of pectin solutions: Fetească Neagră pectin (FNP), Rară Neagră pectin (RNP), commercial apple and citrus pectin (CAP and CCP).

The structural morphology of the pectin extracted by MAE from grape pomace (FN and RN) and commercial pectin samples (apple and citrus) was analysed by scanning electron microscopy (SEM). As Fig. 8 shows, the FN and RN pectin samples obtained by MAE appeared to be very different from commercial apple and citrus pectin, having a coarse and slightly ruptured surface. The structure appeared to be influenced by the accelerated enhancement of temperature and the high internal pressure associated with MAE method^46,87^; similar results in terms of morphology were obtained by Liew et al.^46^. Moreover, microwave irradiation causes a great disintegration in the structural morphology of the raw material, which generates an increase of pectin yield^55,87^. The structure of commercial citrus pectin and grape pomace pectin (FN and RN) was found to have a large number of irregular particles with a rough surface (Fig. 8). This may due to the fact that citrus and grape pomace pectin are rich in insoluble fibres (e.g., lignin, cellulose and hemicellulose); similar structural morphologies have been noted in dried pomace from different vegetables and fruits^25,88^. There are some precise differences in the structure of the samples; the commercial apple pectin showed a slight tendency to curl, while the citrus and grape pomace pectin samples seemed to be ruptured. It was also noticed a more homogenous distribution of particle sizes, an increasing number and size of cavities in the structure of the MAE samples (FN and RN pectin), which could be due to pressure rise and sharp intracellular temperature^55^. From the obtained results, it can be concluded that MAE technique influenced the surface morphology of pectin samples extracted from grape pomace.

## Conclusions

Pectin was extracted from grape pomace pectin (FN and RN) by MAE using three independent variables, each at three levels, microwave power (280, 420 and 560 W), irradiation time (60, 90 and 120 s) and pH (1, 2 and 3). The microwave power applied for pectin extraction and pH of solution were found to have a high impact on all four responses (extraction yield, GalA content, DE and M_w_), while irradiation time had a great influence on pectin yield, GalA content and DE. The optimal conditions for pectin extraction were 560 W, pH of 1.8 for 120 s. The pectin sample extracted by MAE under optimal conditions were compared to CAP and CCP by FT-IR, rheological analysis, DSC and SEM. The viscosity of RN pectin extracted by MAE had a higher viscosity than viscosity of other samples. The microstructure of the pectin samples appeared to be very different from commercial apple and citrus pectin. The grape pomace was found to be a relevant and unconventional source of pectin with a high GalA content, DE and M_w_. The physicochemical properties, morphological characteristics and rheological behavior of pectin extracted by MAE from grape pomace denoted a promising field of different applications of this fiber in food industry.

## Data Availability

The datasets used and/or analyzed during the current study available from the corresponding author on reasonable request.

## References

[1] Dranca F, Oroian M (2018). Extraction, purification and characterization of pectin from alternative sources with potential technological applications. Food Res. Int..

[2] Dalal N, Phogat N, Bisht V, Dhakar U (2020). Potential of fruit and vegetable waste as a source of pectin. Int. J. Chem. Stud..

[3] Hennessey-Ramos L, Murillo-Arango W, Vasco-Correa J, Paz Astudillo IC (2021). Enzymatic extraction and characterization of pectin from cocoa pod husks (*Theobroma cacao* L.) using Celluclast® 1.5 L. Molecules.

[4] Chan S-Y, Choo W-S (2013). Effect of extraction conditions on the yield and chemical properties of pectin from cocoa husks. Food Chem..

[5] Sengar AS, Rawson A, Muthiah M, Kalakandan SK (2020). Comparison of different ultrasound assisted extraction techniques for pectin from tomato processing waste. Ultrason. Sonochem..

[6] Yang J-S, Mu T-H, Ma M-M (2018). Extraction, structure, and emulsifying properties of pectin from potato pulp. Food Chem..

[7] Méndez DA, Fabra MJ, Gómez-Mascaraque L, López-Rubio A, Martinez-Abad A (2021). Modelling the extraction of pectin towards the valorisation of watermelon rind waste. Foods.

[8] Guo Z (2021). Utilization of watermelon peel as a pectin source and the effect of ultrasound treatment on pectin film properties. LWT.

[9] Jiang Y, Xu Y, Li F, Li D, Huang Q (2020). Pectin extracted from persimmon peel: A physicochemical characterization and emulsifying properties evaluation. Food Hydrocoll..

[10] Spinei M, Oroian M (2021). Extraction temperature and pH as decisive factors for the yield and purity of grape pomace pectin. Food Environ. Saf. J..

[11] Colodel C, Vriesmann LC, Teófilo RF, de Oliveira Petkowicz CL (2020). Optimization of acid-extraction of pectic fraction from grape (*Vitis vinifera* cv. Chardonnay) pomace, a Winery Waste. Int. J. Biol. Macromol..

[12] Adetunji LR, Adekunle A, Orsat V, Raghavan V (2017). Advances in the pectin production process using novel extraction techniques: A review. Food Hydrocoll..

[13] Marić M (2018). An overview of the traditional and innovative approaches for pectin extraction from plant food wastes and by-products: Ultrasound-, microwaves-, and enzyme-assisted extraction. Trends Food Sci. Technol..

[14] Thu Dao TA, Webb HK, Malherbe F (2021). Optimization of pectin extraction from fruit peels by response surface method: Conventional versus microwave-assisted heating. Food Hydrocoll..

[15] Cui J (2020). The structure–property relationships of acid- and alkali-extracted grapefruit peel pectins. Carbohydr. Polym..

[16] Cui J (2020). Alkali + cellulase-extracted citrus pectins exhibit compact conformation and good fermentation properties. Food Hydrocoll..

[17] Abou-Elseoud WS, Hassan EA, Hassan ML (2021). Extraction of pectin from sugar beet pulp by enzymatic and ultrasound-assisted treatments. Carbohydr. Polym. Technol. Appl..

[18] Dranca F, Vargas M, Oroian M (2020). Physicochemical properties of pectin from Malus domestica ‘Fălticeni’ apple pomace as affected by non-conventional extraction techniques. Food Hydrocoll..

[19] Karbuz P, Tugrul N (2021). Microwave and ultrasound assisted extraction of pectin from various fruits peel. J. Food Sci. Technol..

[20] Mao G (2019). Reconsidering conventional and innovative methods for pectin extraction from fruit and vegetable waste: Targeting rhamnogalacturonan I. Trends Food Sci. Technol..

[21] Liew SQ, Teoh WH, Tan CK, Yusoff R, Ngoh GC (2018). Subcritical water extraction of low methoxyl pectin from pomelo (*Citrus grandis* (L.) Osbeck) peels. Int. J. Biol. Macromol..

[22] Wang S (2007). Optimization of pectin extraction assisted by microwave from apple pomace using response surface methodology. J. Food Eng..

[23] Prakash Maran J, Sivakumar V, Thirugnanasambandham K, Sridhar R (2013). Optimization of microwave assisted extraction of pectin from orange peel. Carbohydr. Polym..

[24] Maran JP, Swathi K, Jeevitha P, Jayalakshmi J, Ashvini G (2015). Microwave-assisted extraction of pectic polysaccharide from waste mango peel. Carbohydr. Polym..

[25] Rodsamran P, Sothornvit R (2019). Microwave heating extraction of pectin from lime peel: Characterization and properties compared with the conventional heating method. Food Chem..

[26] Filisetti-Cozzi TMCC, Carpita NC (1991). Measurement of uronic acids without interference from neutral sugars. Anal. Biochem..

[27] Melton, L. D. & Smith, B. G. Determination of the uronic acid content of plant cell walls using a colorimetric assay. *Curr. Protoc. Food Anal. Chem.* (2001).

[28] Miceli-Garcia LG (2014). Pectinfrom Apple Pomace: Extraction, Characterization, and Utilization in Encapsulating Alpha-Tocopherol Acetate.

[29] Dranca F, Oroian M (2019). Ultrasound-assisted extraction of pectin from Malus domestica ‘Fălticeni’ apple pomace. Processes.

[30] Franchi, M. L. Evaluation of enzymatic pectin extraction by a recombinant polygalacturonase (PGI) from apples and pears pomace of argentinean production and characterization of the extracted pectin. *J. Food Process. Technol.* **05**, (2014).

[31] Wai WW, Alkarkhi AFM, Easa AM (2010). Effect of extraction conditions on yield and degree of esterification of durian rind pectin: An experimental design. Food Bioprod. Process..

[32] Wang W (2016). Characterization of pectin from grapefruit peel: A comparison of ultrasound-assisted and conventional heating extractions. Food Hydrocoll..

[33] Bagherian H, Zokaee Ashtiani F, Fouladitajar A, Mohtashamy M (2011). Comparisons between conventional, microwave- and ultrasound-assisted methods for extraction of pectin from grapefruit. Chem. Eng. Process. Process Intensif..

[34] Guo X (2012). Extraction of pectin from navel orange peel assisted by ultra-high pressure, microwave or traditional heating: A comparison. Carbohydr. Polym..

[35] Hosseini SS, Khodaiyan F, Yarmand MS (2016). Optimization of microwave assisted extraction of pectin from sour orange peel and its physicochemical properties. Carbohydr. Polym..

[36] Maran JP, Prakash KA (2015). Process variables influence on microwave assisted extraction of pectin from waste *Carcia papaya* L. peel. Int. J. Biol. Macromol..

[37] Swamy GJ, Muthukumarappan K (2017). Optimization of continuous and intermittent microwave extraction of pectin from banana peels. Food Chem..

[38] Seixas FL (2014). Extraction of pectin from passion fruit peel (*Passiflora edulis* f. flavicarpa) by microwave-induced heating. Food Hydrocoll..

[39] Košťálová Z, Aguedo M, Hromádková Z (2016). Microwave-assisted extraction of pectin from unutilized pumpkin biomass. Chem. Eng. Process. Process Intensif..

[40] Thirugnanasambandham K, Sivakumar V, Prakash Maran J (2014). Process optimization and analysis of microwave assisted extraction of pectin from dragon fruit peel. Carbohydr. Polym..

[41] Hosseini SS, Khodaiyan F, Yarmand MS (2016). Aqueous extraction of pectin from sour orange peel and its preliminary physicochemical properties. Int. J. Biol. Macromol..

[42] Yoo S-H (2012). Structural characteristics of pumpkin pectin extracted by microwave heating. J. Food Sci..

[43] Lefsih K (2017). Pectin from *Opuntia ficus* indica: Optimization of microwave-assisted extraction and preliminary characterization. Food Chem..

[44] Rahmani Z, Khodaiyan F, Kazemi M, Sharifan A (2020). Optimization of microwave-assisted extraction and structural characterization of pectin from sweet lemon peel. Int. J. Biol. Macromol..

[45] Mellinas C, Ramos M, Jiménez A, Garrigós MC (2020). Recent trends in the use of pectin from agro-waste residues as a natural-based biopolymer for food packaging applications. Materials (Basel).

[46] Liew SQ, Ngoh GC, Yusoff R, Teoh WH (2016). Sequential ultrasound-microwave assisted acid extraction (UMAE) of pectin from pomelo peels. Int. J. Biol. Macromol..

[47] Thirugnanasambandham K, Sivakumar V (2015). Application of D-optimal design to extract the pectin from lime bagasse using microwave green irradiation. Int. J. Biol. Macromol..

[48] Prakash Maran J, Sivakumar V, Thirugnanasambandham K, Sridhar R (2014). Microwave assisted extraction of pectin from waste *Citrullus lanatus* fruit rinds. Carbohydr. Polym..

[49] Najari Z, Khodaiyan F, Yarmand MS, Hosseini SS (2022). Almond hulls waste valorization towards sustainable agricultural development: Production of pectin, phenolics, pullulan, and single cell protein. Waste Manag..

[50] Sayah MY (2016). Yield, esterification degree and molecular weight evaluation of pectins isolated from orange and grapefruit peels under different conditions. PLoS ONE.

[51] Li D, Jia X, Wei Z, Liu Z (2012). Box-Behnken experimental design for investigation of microwave-assisted extracted sugar beet pulp pectin. Carbohydr. Polym..

[52] Shen S (2022). Structures, physicochemical and bioactive properties of polysaccharides extracted from Panax notoginseng using ultrasonic/microwave-assisted extraction. LWT.

[53] Kute AB, Mohapatra D, Kotwaliwale N, Giri SK, Sawant BP (2020). Characterization of pectin extracted from orange peel powder using microwave-assisted and acid extraction methods. Agric. Res..

[54] Antonić B, Jančíková S, Dordević D, Tremlová B (2020). Grape pomace valorization: a systematic review and meta-analysis. Foods.

[55] Zheng J (2021). Radio frequency assisted extraction of pectin from apple pomace: Process optimization and comparison with microwave and conventional methods. Food Hydrocoll..

[56] Oh SY, Yoo D, Shin Y, Seo G (2005). FTIR analysis of cellulose treated with sodium hydroxide and carbon dioxide. Carbohydr. Res..

[57] Sivashankari PR, Krishna Kumar K, Devendiran M, Prabaharan M (2021). Graphene oxide-reinforced pectin/chitosan polyelectrolyte complex scaffolds. J. Biomater. Sci. Polym. Ed..

[58] Chaharbaghi E, Khodaiyan F, Hosseini SS (2017). Optimization of pectin extraction from pistachio green hull as a new source. Carbohydr. Polym..

[59] Tian L, Zhao Y, Guo C, Yang X (2011). A comparative study on the antioxidant activities of an acidic polysaccharide and various solvent extracts derived from herbal *Houttuynia cordata*. Carbohydr. Polym..

[60] Khalili P, Tshai KY, Kong I (2018). Comparative thermal and physical investigation of chemically treated and untreated oil palm EFB fiber. Mater. Today Proc..

[61] Yu T, Jiang N, Li Y (2014). Study on short ramie fiber/poly(lactic acid) composites compatibilized by maleic anhydride. Compos. Part A Appl. Sci. Manuf..

[62] González Moreno A (2021). Pectin-cellulose nanocrystal biocomposites: Tuning of physical properties and biodegradability. Int. J. Biol. Macromol..

[63] Shechter M, Chefetz B (2008). Insights into the sorption properties of cutin and cutan biopolymers. Environ. Sci. Technol..

[64] Coimbra P (2011). Preparation and chemical and biological characterization of a pectin/chitosan polyelectrolyte complex scaffold for possible bone tissue engineering applications. Int. J. Biol. Macromol..

[65] Meor Hussin AS (2013). Characterisation of lignocellulosic sugars from municipal solid waste residue. Biomass Bioenergy.

[66] La Torre C, Caputo P, Plastina P, Cione E, Fazio A (2021). Green husk of walnuts (*Juglans regia* L.) from Southern Italy as a valuable source for the recovery of glucans and pectins. Fermentation.

[67] Guo, Q., Ai, L. & Cui, S. W. Fourier transform infrared spectroscopy (FTIR) for carbohydrate analysis. in *Methodology for Structural Analysis of Polysaccharides* 69–71 (2018). 10.1007/978-3-319-96370-9_9.

[68] Kacuráková M (2000). FT-IR study of plant cell wall model compounds: pectic polysaccharides and hemicelluloses. Carbohydr. Polym..

[69] Szymanska-Chargot M, Zdunek A (2013). Use of FT-IR spectra and PCA to the bulk characterization of cell wall residues of fruits and vegetables along a fraction process. Food Biophys..

[70] Yu M (2021). Effects of different extraction methods on structural and physicochemical properties of pectins from finger citron pomace. Carbohydr. Polym..

[71] Nisar T (2018). Characterization of citrus pectin films integrated with clove bud essential oil: Physical, thermal, barrier, antioxidant and antibacterial properties. Int. J. Biol. Macromol..

[72] Ezzati S, Ayaseh A, Ghanbarzadeh B, Heshmati MK (2020). Pectin from sunflower by-product: Optimization of ultrasound-assisted extraction, characterization, and functional analysis. Int. J. Biol. Macromol..

[73] Einhorn-Stoll U, Kunzek H (2009). The influence of the storage conditions heat and humidity on conformation, state transitions and degradation behaviour of dried pectins. Food Hydrocoll..

[74] Einhorn-Stoll U, Kunzek H, Dongowski G (2007). Thermal analysis of chemically and mechanically modified pectins. Food Hydrocoll..

[75] Wang X, Chen Q, Lü X (2014). Pectin extracted from apple pomace and citrus peel by subcritical water. Food Hydrocoll..

[76] Priyangini F, Walde SG, Chidambaram R (2018). Extraction optimization of pectin from cocoa pod husks (*Theobroma cacao* L.) with ascorbic acid using response surface methodology. Carbohydr. Polym..

[77] Lewandowska, K., Dąbrowska, A. & Kaczmarek, H. Rheological properties of pectin, poly(vinyl alcohol) and their blends in aqueous solutions. *e-Polymers* **12** (2012).

[78] Chan SY, Choo WS, Young DJ, Loh XJ (2017). Pectin as a rheology modifier: Origin, structure, commercial production and rheology. Carbohydr. Polym..

[79] Min B (2011). Environmentally friendly preparation of pectins from agricultural byproducts and their structural/rheological characterization. Bioresour. Technol..

[80] Sousa AG, Nielsen HL, Armagan I, Larsen J, Sørensen SO (2015). The impact of rhamnogalacturonan-I side chain monosaccharides on the rheological properties of citrus pectin. Food Hydrocoll..

[81] Vriesmann LC, Teófilo RF, Lúcia de Oliveira Petkowicz C (2012). Extraction and characterization of pectin from cacao pod husks (*Theobroma cacao* L.) with citric acid. LWT.

[82] Padmanabhan PA, Kim D-S, Pak D, Sim SJ (2003). Rheology and gelation of water-insoluble dextran from Leuconostoc mesenteroides NRRL B-523. Carbohydr. Polym..

[83] Li G (2022). Physicochemical, structural and rheological properties of pectin isolated from citrus canning processing water. Int. J. Biol. Macromol..

[84] Chen Y, Zhang J-G, Sun H-J, Wei Z-J (2014). Pectin from Abelmoschus esculentus: Optimization of extraction and rheological properties. Int. J. Biol. Macromol..

[85] Barbieri SF (2019). Pectins from the pulp of gabiroba (*Campomanesia xanthocarpa* Berg): Structural characterization and rheological behavior. Carbohydr. Polym..

[86] Iagher F, Reicher F, Ganter JLM (2002). Structural and rheological properties of polysaccharides from mango (*Mangifera indica* L.) pulp. Int. J. Biol. Macromol..

[87] Kazemi M, Khodaiyan F, Hosseini SS (2019). Utilization of food processing wastes of eggplant as a high potential pectin source and characterization of extracted pectin. Food Chem..

[88] Lal AMN (2021). Pulsed electric field combined with microwave-assisted extraction of pectin polysaccharide from jackfruit waste. Innov. Food Sci. Emerg. Technol..

